# Automated nutrient screening system enables high-throughput optimisation of microalgae production conditions

**DOI:** 10.1186/s13068-015-0238-7

**Published:** 2015-04-11

**Authors:** Khairul Adzfa Radzun, Juliane Wolf, Gisela Jakob, Eugene Zhang, Evan Stephens, Ian Ross, Ben Hankamer

**Affiliations:** Institute for Molecular Bioscience, The University of Queensland, St Lucia, Queensland 4072 Australia; Faculty of Applied Sciences, Universiti Teknologi MARA, Shah Alam, 40450 Selangor Malaysia

**Keywords:** Algae, Nutrient, Biomass, Screening, High-throughput, Growth

## Abstract

**Background:**

Microalgae provide an excellent platform for the production of high-value-products and are increasingly being recognised as a promising production system for biomass, animal feeds and renewable fuels.

**Results:**

Here, we describe an automated screen, to enable high-throughput optimisation of 12 nutrients for microalgae production. Its miniaturised 1,728 multiwell format allows multiple microalgae strains to be simultaneously screened using a two-step process. Step 1 optimises the primary elements nitrogen and phosphorous. Step 2 uses *Box-Behnken* analysis to define the highest growth rates within the large multidimensional space tested (Ca, Mg, Fe, Mn, Zn, Cu, B, Se, V, Si) at three levels (−1, 0, 1). The highest specific growth rates and maximum OD_750_ values provide a measure for continuous and batch culture.

**Conclusion:**

The screen identified the main nutrient effects on growth, pairwise nutrient interactions (for example, Ca-Mg) and the best production conditions of the sampled statistical space providing the basis for a targeted full factorial screen to assist with optimisation of algae production.

**Electronic supplementary material:**

The online version of this article (doi:10.1186/s13068-015-0238-7) contains supplementary material, which is available to authorized users.

## Background

Microalgae provide the biotechnology industry with an excellent platform for the production of a broad range of high-value products (for example, pigments, unsaturated fatty acids and expressed proteins), animal and aquaculture feeds and also represent a rapidly advancing technology for the production of biocommodities like biomass feedstocks for renewable fuel production (for example, oil-based fuels, methane, alcohols and hydrogen). Increasing the economic viability of production requires process optimisation, and a key aspect of this is maximising biomass quality and yield (that is, maximum growth rate and total biomass concentration).

The development of improved growth media for microalgae is an important aspect of this strategy. The significance of nutrient optimisation is highlighted by the fact that microalgae have adapted to a diverse range of environments (for example, varying C, N and P source and concentration) and so are likely to have different nutrient requirements. The bioavailability of each respective element also depends significantly on factors such as solubility, chemical speciation (for example, Fe^2+^ and Fe^3+^), pH, ionic strength, inorganic anions, chelates or interaction with other elements (for example, the formation of Ca_3_(PO_4_)_2_ precipitates). Nutrients must therefore be supplied in a bioavailable form, and while nutrient deficiencies can limit culture growth and/or health, the supply of excess nutrients can result in nutrient wastage (for example, opportunistic P uptake), toxic effects and additional waste streams. Nutrient optimisation therefore impacts economic and environmental sustainability as well as the overall energy balance of the system.

Given the broad biodiversity of microalgae and the wide range of conditions that they grow under, it is not surprising that their elemental composition varies. Carbon is reported to be the most abundant element in biomass (approximately 40% to 60% of the ash-free biomass) being a component of most biomolecules, and can be provided in the form of CO_2_ (for photoautotrophic, photoheterotrophic and mixotrophic growth regimes) and/or organic carbon forms such as acetate (for mixotrophic, photoheterotrophic and heterotrophic growth modes). Oxygen, also a component of most biomolecules, is thought to be the next most abundant element (for example, approximately 12% to 29%) [1] of biomass and is an integral part of most biomass components including proteins, carbohydrates and oils. The macroelements N (approximately 7% of biomass), S and P (approximately 1% of biomass) [2] are mainly involved in protein and nucleic acid synthesis as well as regulatory pathways in the cell and are indispensible. Mg is a major component of chlorophylls and a co-factor for enzymes while Ca is part of the water oxidizing complex of photosystem II an important element in the CO_2_ fixation process and involved in ion transport and intracellular signalling [3]. K, Na and Cl are prominent in the cytoplasm and play key roles in osmoregulation [4]. Microelements (trace elements) are also often required as co-factors (for example, for enzymes and cell signalling). They include Fe, Cu, Mn, Mo and V which can exist in multiple stable oxidation states and so take part in redox chemistry [5]. Mo and V are relatively soluble as oxy-anions and are important co-factors in enzymes of S and N metabolism [5]. Zn and Co provide additional catalytic capacity. Zn acts as a co-factor in many enzymes and supports the function of transcription factors, and its concentration may be regulated in part by metallothioneins [6]. Co is a constituent of both of vitamin B_1_ and B_12_ co-enzymes, which are involved in a range of metabolic reactions [5-7]. Silicon is an abundant element but is not generally found at high levels in biology due to the insolubility of its oxides or hydroxides. Nevertheless, Si is important for specific algae (such as diatoms) that build up hard Si-rich cell walls (frustules) [8]. Fe is also an essential micronutrient for all living organisms primarily because it can efficiently accept and donate electrons (for example, reduction/oxidation of Fe-S clusters). In this context, Fe plays a crucial role in electron transport processes such as respiration and photosynthesis. Fe-containing proteins are also directly involved in nitrate and nitrite reduction, chlorophyll synthesis and the detoxification of O_2_ radicals [9,10]. In summary then, each of these elements will be required in a suitable concentration range and bioavailable form and the optimisation of these conditions has been conducted in agriculture and horticulture over centuries.

In terms of algae production, growth media have until now largely been based on the following:

1. The results of elemental analysis of biomass [11]
2. Flask scale trials and complete statistical screens of selected variable (for example, N and P) [12].

Both of these approaches are theoretically suboptimal. First, basing media formulations on elemental analysis of biomass is no guarantee that optimal conditions will be achieved, as the original biomass analysed, may itself have been produced under suboptimal conditions such as nutrient limitation or excess. Second, the use of complete factorial analysis of a few selected variables fails to identify the theoretical optimum of production. For example, single nutrient analysis (for example, of N) misses the role of potential interactions between other nutrients (for example, Ca and P) [5,11-15], and furthermore, micronutrient requirements may vary under different growth conditions.

A third approach involves the use of a full factorial screen. This would provide a statistically valid analysis but is limited by the fact that the optimisation of the 21 most commonly used macro- (C, N, P, K, Ca, Mg, S, Na, Cl) and microelements (Fe, Mn, Zn, Cu, B, Mo, Si, Se, V, Co, Ni, I) (excluding additional commonly used amino acids, vitamins and/or other additives) at even three different concentrations would require 3^21^ experiments = 10,460,353,203). Clearly, this is an impractically large set of variables to analyse which would have to be increased further if proper consideration was given to nitrogen type (for example, NO_3_^-^, NH_4_^+^ or urea), different carbon forms (for example, glucose or acetate) to support photoheterotrophic, mixotrophic or heterotrophic growth and other supplements (for example, vitamins). Yet despite this, failure to analyse this full and complex statistical space is likely to result in the inability to identify optimal conditions.

Here, we report an advanced miniaturised high-throughput robotic screen designed to identify the best nutrient conditions within this complex multidimensional statistical space and is suitable to analyse a broad range of microalgae species. The screen contains the above 21 mineral elements and vitamins B_1_ and B_12_ and focuses on the statistical optimisation of 12 of the most important of these. These include the 12 macro- (N (that is, NO_3_^−^, NH_4_^+^ and urea), P, Ca, Mg) and microelements (Mn, Zn, Cu, B, V, Si, Fe, Se) with the remaining nutrients provided in reportedly replete levels at 1% CO_2_ concentration (near optimal, though this can be adjusted).

**Figure 1:**
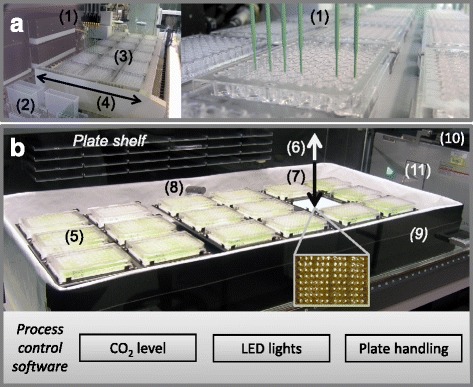
The screening system used for nutrient optimisation. **(a)** Automated media preparation equipment and **(b)** automated growth chamber. See ‘Material and methods’ for details.

Through the use of an automated two-phase screening process (Step 1: optimising N and P; Step 2: all other variables) and the use of an incomplete factorial *Box-Behnken* design (10 elements at 3 concentrations = 3^10^ = 59,049 full factorial conditions), the statistical search space was compressed over 328-fold to 180 trials in Step 2. A total of 246 trials (for Steps 1 and 2) were performed for each strain analysed. Specifically, the system is designed to measure growth curves based on optical density at 750 nm (OD_750_, a proxy measure for biomass) to measure maximum growth rates (as a model for continuous culture) and total biomass yield (as a model of batch culture) (Figure 1). The measurement of growth rate was the usual approach, and based on this, the statistical performance of each condition was evaluated to identify,

1. the best nutrient mix for a given algae cell line,
2. the best concentration range for each nutrient (that is, limiting, sufficient and toxic levels of supply) which has operational importance for scale up, and
3. positive and negative statistical nutrient interactions that affect microalgae biomass production.

The screen therefore provides an excellent basis for next phase targeted full factorial screens under scaled up conditions on the path to commercial process optimisation for efficient biomass production.

## Results and discussion

### Two-step nutrient screening matrix design

**Figure 2:**
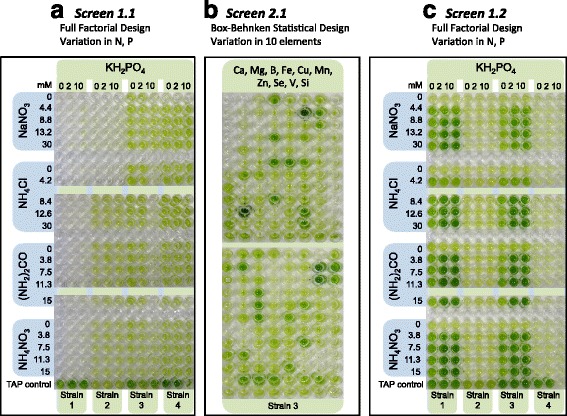
The multidimensional nutrient screen. **(a)** Initial Screen 1 (Screen 1.1) optimises phosphate concentration (0, 2, 10 mM KH_2_PO_4_) as well as N type (NaNO_3_, NH_4_Cl, (NH_2_)_2_CO and NH_4_NO_3_) and concentration (0 to 30 mM). The N source concentration is adjusted to account for the number of N atoms in the source (for example, NaNO_3_ = 1, NH_4_NO_3_ = 2). **(b)** Screen 2 uses a *Box-Behnken* design in which the nitrogen/phosphate condition that yielded the highest growth rate in Screen 1 form the midpoint of this multidimensional screen. It has three concentration levels (−1, 0 and +1) for the elements Ca, Mg, Fe, Cu, Mn, Zn, B, Se, V and Si. The trace elements Mo, Co and vitamins B_1_ and B_12_ are kept constant (see ‘Materials and methods’). Screens 1 and 2 are designed to measure the rate of change in optical density (for example, OD_750_) to define specific growth rates and total yield as a proxy for biomass production. **(c)** Screen 1.2 is the repeat of Screen 1.1 with adjusted concentrations of the elements tested in Screen 2 to demonstrate algae performance improvements.

The layouts of the Screens 1 and 2 matrices used to identify the best nutrient mixtures for a single species are shown in Figure 2a, b, respectively. The Screen 1 ‘*matrix unit*’ consists of 63 microwells. This *matrix unit* was copied 24 times across the eighteen 96-well plates to construct the full *‘24 unit Screen 1 matrix*’ that formed a full 1,512-well screen run (Figure 1b). The *24 unit Screen 1 matrix* enables 24 individual species (or 8 species in triplicate) to be analysed simultaneously.

The Screen 2 ‘*matrix unit*’ consists of 183 microwells (180 screen solutions and 3 internal Tris-acetate-phosphate (TAP) controls). TAP is a standard medium for chlorophytes and contains acetate which can be metabolised by most microalgae, thereby allowing mixotrophic growth. Mixotrophic growth generally yields higher growth rates than photoautotrophic growth providing a positive control. It was copied 9 times across the eighteen 96-well plates to construct the full ‘*9 unit Screen 2 matrix*’ that formed a full 1,647-well screen run (Figure 1b). The *9 unit Screen 2 matrix* enables 9 individual species (or 3 species in triplicate) to be analysed simultaneously.

The full factorial Screen 1 (Figure 2a) consists of three phosphate (KH_2_PO_4_) concentrations (0, 2 and 10 mM) and five nitrogen concentrations for NaNO_3_ (to identify NO_3_^−^ utilising strains), NH_4_Cl (to identify NH_4_^+^ utilising strains), NH_4_NO_3_ (to identify NH_4_^+^ and NO_3_^−^ utilising strains) and urea (NH_2_)_2_CO (to identify strains that can use this alternative cheaper nitrogen source). The values of 0, 2 and 10 mM KH_2_PO_4_ were selected to provide phosphate-limited conditions (0 mM), an average literature value (2 mM) (Additional file 1: Table S3) and excess phosphate (10 mM) (see ‘Materials and methods’). A similar approach was taken to set the N concentrations in Screen 1, for the N sources NaNO_3_, NH_4_Cl, NH_4_NO_3_ and (NH_2_)_2_CO. All other nutrients were set as described in Table 1 to reflect average literature values.

**Table 1:** **Nutrient formulation of the Screen 1 system**

**Nutrient category**	**Nutrient**	**Final concentration (mM)**
Nitrogen	NH_4_Cl	0, 4.2, **8.4**, 12.6, 30
	NaNO_3_	0, 4.4, **8.8**, 13.2, 30
	(NH_2_)_2_CO	0, 3.8, **7.5**, 11.3, 15
	NH_4_NO_3_	0, 3.8, **7.5,** 11.3, 15
Phosphate	KH_2_PO_4_	0, **2**, 10
Macroelements	CaCl_2_ · 2H_2_O	**0.213** *(0.85)*
	MgSO_4_ · 7H_2_O^a^	**0.375** *(1.50)*
Microelements	H_3_BO_3_^a^	0.184
	Fe_2_(SO_4_)_3_ · 7H_2_O (*^1^)^a^	0.001
	CuSO_4_ · 5H_2_O^a^	0.0064
	MnCl_2_ · H_2_O^a^	0.0258
	ZnSO_4_ · 7H_2_O^a^	0.077
	Na_2_SeO_3_^a^	0.0001
	VOSO_4_ · H_2_O^a^	0.000009
	Na_2_SiO_3_ · 5H_2_O^a^	0.273
	(NH_4_)_6_MoO_4_ · 4H_2_O^a^	0.00089
	CoCl_2_ · 6H_2_O^a^	0.0067
Chelating agent	Na_2_EDTA, pH 8.0	0.5373
Buffer	Tris-HCl, pH 7.4	100
Vitamins	Vitamin B_1_ (thiamine hydrochloride)	**0.052**
	Vitamin B_12_ (cyanocobalamin)	**0.0001**

The average nutrient concentrations based on the literature analysis (Additional file 1: Table S3) are indicated in bold fonts. Refined nutrient concentrations after iterative cycling of nutrient screens are indicated in italic fonts. ^a^Nutrient elements were prepared as *Basal Medium* for 5000 tests. (*^1^) Fe_2_(SO_4_)_3_ · 7H_2_O is prepared in 0.5360 mM Na_2_-EDTA, pH 8.

### Preliminary screening using macronutrients

Eight randomly selected algae strains were screened (*Micractinium inermum* (18-1), *Ankistrodesmus gracilis* (18-2), *Rhombocystis complanata* (SF-150), *Chlorella sorokiniana* (21), *Monoraphidium convolutum* (*9-FW*), *C. pyrenoidosa* (22), *M. reisseri* (13), *Podohedriella falcate* (4A-1)) using a preliminary Screen 1 (Screen 1.1). The results of four of these are shown as a photographic profile after 72 h of incubation in Figure 2a. The growth rate in all of the photoautotrophic conditions tested was very much lower than that of the TAP control. As CO_2_ was provided as a 1% CO_2_:Air (*v*/*v*) mixture, this suggested that nutrient(s) other than CO_2_, N and P might be present either in limiting or inhibitory concentrations despite being based on average literature values.

### Growth curve fitting

**Figure 3:**
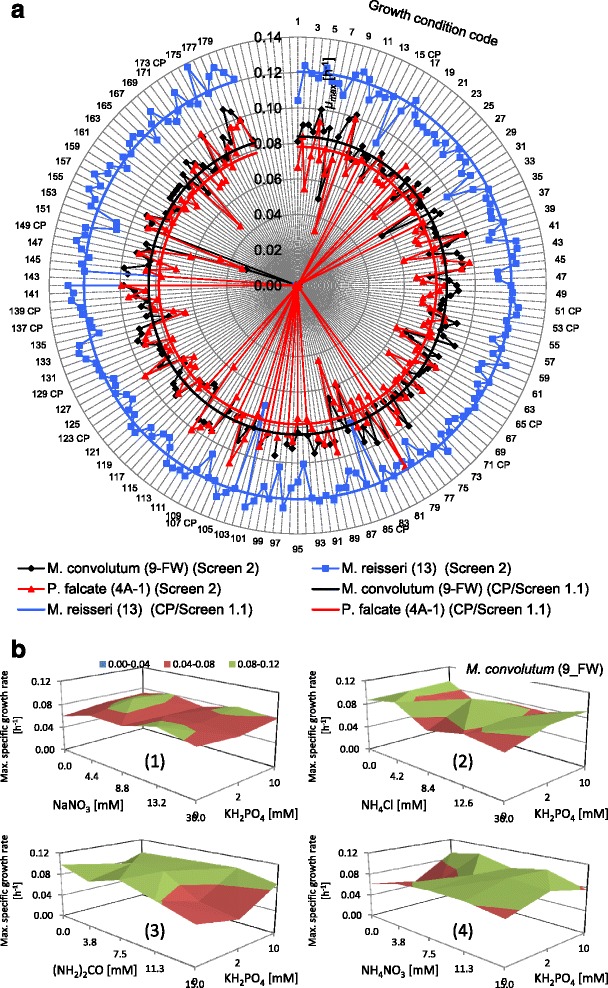
Radial plots of the highest specific growth rates of *M. convolutum* (9-FW) (black), *M. reisseri* (13) (blue) and *P. falcate* (4A-1) (red) in 180 Screen 2 conditions (a) and growth rate analysis for *M. convolutum* (9-FW) in the subsequent Screen 1.2 (b). **(a)** The radial plot displays the highest specific growth rate *μ*_max_ (h^−1^) for each condition indicated by the distance of a given data point from the centre of the plot. The radial plot shows growth rate data of Screen 1*.*1 in comparison to Screen 2 to visualise the growth performance improvement. The growth rate performance of three key strains in Screen 2 was much higher than in Screen 1*.*1. **(b)** Surface chart of the growth rate data analysis in Screen 1.2. The growth rate data are shown as a function of the nitrogen (N) and phosphate (P) concentration exemplary for *M. convolutum* (9_FW) in individual plots for media containing NaNO_3_ (1), NH_4_Cl (2), (NH_2_)_2_CO (3) and NH_4_NO_3_ (4) as nitrogen source.

**Figure 4:**
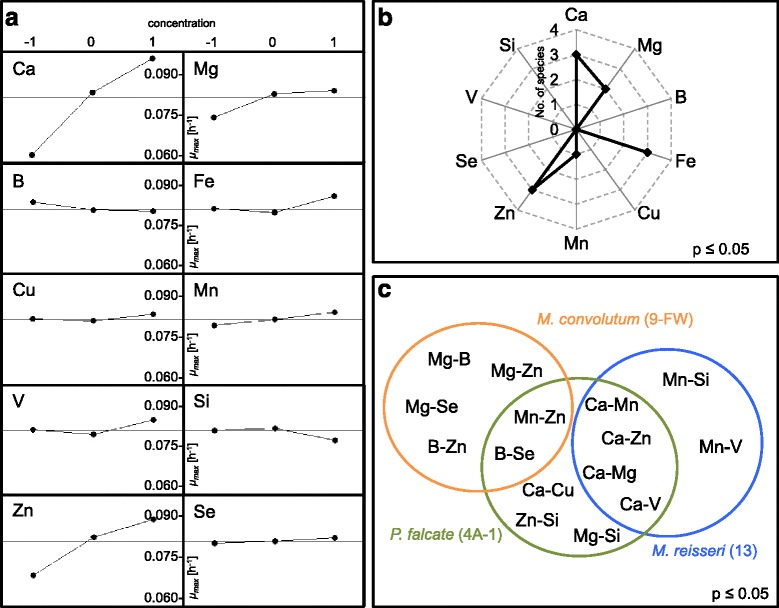
Main and Interaction Effects analysis. **(a)** Summary plot of the *Main Effects p* value analysis for *M. convolutum* (9-FW) exhibiting significant nutrient effects on microalgae growth rates (h^−1^). **(b)** Radial plot showing the number of microalgae strains of the eight tested that exhibited significant *Main Effects p* value for specific elements. Three strains exhibited significant *Main Effects p* value for calcium, two strains exhibited significant *Main Effects p* value for magnesium. Overall, calcium, magnesium, iron, zinc and manganese were found to be significant and require modulation for improved growth to be achieved. **(c)** Venn diagram showing pairwise interactions identifying a number of potential species/nutrient mix specific effects (for example, Mg-Zn in *M. convolutum* (9_FW)) or interactions common to more than one species (for example, Ca-Mg for *P. falcate* and *M. reisseri)*.

**Figure 5:**
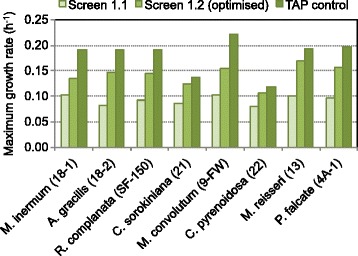
Improvement of photoautotrophic growth performance. Histogram showing the highest growth rate of the microalgae strains *M. inermum* (18-1), *A. gracilis* (18-2), *R. complanata* (SF-150), *C. sorokiniana* (21), *M. convolutum* (*9-FW*), *C. pyrenoidosa* (22), *M. reisseri* (13) and *P. falcate* (4A-1) in photoautotrophic (Screen 1.1, Screen 1.2) and in photoheterotrophic conditions (TAP medium).

**Figure 6:**
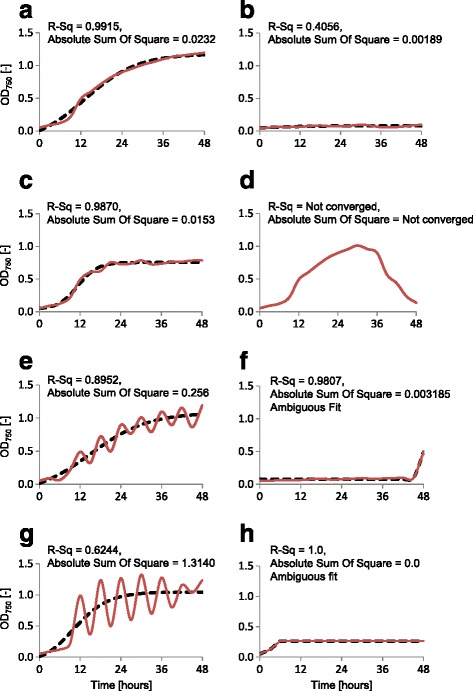
Data analysis and quality control. Examples (**a-h**) of common microalgal growth curve morphologies (red) and modelled fits calculated using *GraphPad Prism* (black). Growth curve patterns that must be accounted for include **(a)** Robust growth. **(b)** No growth. **(c)** Growth with lower maximum OD. **(d)** Cell growth, followed by cell death. **(e)** Growth Intermediate circadian rhythm. **(f)** A long lag phase. **(g)** Strong circadian rhythm. **(h)** Initial growth followed by plateau. The main aim of this curve fitting step is to automate the identification of typical growth curves and eliminate atypical growth curves that could introduce errors into the downstream analysis to provide sigmoidal fits that could be used to determine specific growth rates more accurately.

The parallel acquisition and screening of approximately 1,700 growth curves requires automation and robust quality control to ensure that the maximum specific growth rates are as accurately defined as possible for later statistical analysis (Figures 3, 4 and 5). Figure 6 shows schematic examples of growth curve patterns observed and the quality control processes implemented. Figure 6a shows an example of a typical robust growth curve and the fitted model (*R*^2^ value = 99.15%). The converse‚‘no growth’ example is shown in Figure 6b. Due to light and nutrient limitation even the most rapidly growing cultures plateau at approximately OD_750_ = 1.0. Control experiments (data not shown) confirmed that the plate reader yielded a linear relationship between OD_750_ and biomass within and beyond this range. Conditions in which maximum OD_750_ values less than 0.8 were obtained were checked manually to discriminate between the alternatives of continuous (but slow) growth or rapid growth with an early endpoint limitation due to nutrient depletion. Figure 6c shows an example of a sigmoidal curve with lower maximum OD_750_ and weak strain-specific circadian rhythm. Fitting a sigmoidal curve through the data allows the circadian rhythm component of the model to be eliminated. Figure 6d shows a profile which includes an apparent cell growth and subsequent cell death phase. As a result, a good sigmoidal fit was not possible (*R*^2^ did not converge) and such conditions were eliminated from the final analysis. Figure 6e depicts a growth curve with an intermediate strength circadian rhythm or intermittent flocculation, while Figure 6g shows a stronger circadian rhythm/intermittent flocculation pattern. Figure 6f, h is an example of curves yielding ambiguous fits. These were manually excluded from further analysis based on the low max OD_750_.

### Automated sampling and data analysis

Table 2 shows the three highest growth rates (in bold) obtained for the eight species tested and the N and P conditions under which these were obtained. Closer analysis shows that the relative standard deviation (RSD) is well below 5% and sometimes below 1% between biological replicates. Typically, manual OD measurements yield approximately 10% error levels between biological replicates. The low standard deviations achieved in this automatic screen were due to three factors. First, automating nutrient solution preparation reduced dispensing errors. Second, optical density measurement errors were also reduced through automation. Third, the process of automation enabled the collection of a large number of data points (24 for each experiment) which improved the statistical curve fit used to calculate the *μ*_max_ value for each experiment condition. The RSD values therefore reflect these collective improvements over manual analysis.

**Table 2:** **Summary of specific growth rates of Screen 1.2**

**Strain**	**TAP media,** ***μ***_**max**_ **[h** ^**−1**^ **]**	**Mean** ***μ***_**max**_ **[h** ^**−1**^ **]**	**Highest individual** ***μ***_**max**_ **[h** ^**−1**^ **]**	**N and P source**
*M. inermum* (18-1)	0.195 ± 0.004	0.116 ± 0.001	0.135 ± 0.002	3.8 mM NH_4_NO_3_
				2 mM KH_2_PO_4_
*A. gracilis* (18-2)	0.187 ± 0.008	0.116 ± 0.001	0.147 ± 0.007	3.8 mM NH_4_NO_3_
				10 mM KH_2_PO_4_
*R. complanata* (SF150)	0.197 ± 0.003	0.123 ± 0.003	0.145 ± 0.009	13.2 mM NaNO_3_
				2 mM KH_2_PO_4_
*C. sorokiniana* (21)	0.116 ± 0.001	0.104 ± 0.002	0.125 ± 0.003	3.8 mM NH_4_NO_3_
				2 mM KH_2_PO_4_
***M. convolutum*** ** (9-FW)**	**0.230 ± 0.008**	**0.127 ± 0.004**	**0.156 ± 0.002**	**8.4 mM NH**_**4**_ **Cl**
				**10 mM KH**_**2**_ **PO**_**4**_
*C. pyrenoidosa* (22)	0.116 ± 0.001	0.095 ± 0.001	0.107 ± 0.002	8.4 mM NH_4_Cl
				2 mM KH_2_PO_4_
***M. reisseri *** **(13)**	**0.215 ± 0.003**	**0.144 ± 0.003**	**0.168 ± 0.008**	**3.8 mM (NH**_**2**_ **)**_**2**_ **CO**
				**10 mM KH**_**2**_ **PO**_**4**_
***P. falcate *** **(4A-1)**	**0.191 ± 0.002**	**0.129 ± 0.004**	**0.156 ± 0.007**	**7.5 mM (NH**_**2**_ **)**_**2**_ **CO**
				**2 mM KH**_**2**_ **PO**_**4**_

Algal strains were cultivated in triplicates. The rows in bold represent the best performing algal strains and conditions.

It should be noted that the process of automated OD measurement requires the robotic removal of the lid of each 96-microwell plate within the cultivation chamber for a period of 2.7 min per measurement (once every 3 h). As a result, it is not possible to completely eliminate evaporation. However, the reduction of the optical path length through evaporation is compensated for by the concomitant increase cell concentration. Regardless, to minimise these effects, the *μ*_max_ values were typically calculated during the first 24 to 48 h of the experiment. It is of note that despite these small evaporative losses, the RSD values are significantly lower than can be achieved through manual measurement (see above). As the automation process enables the analysis of 1,728 samples simultaneously and the RSD values are low, it is concluded that it is an acceptable compromise.

### Strain performance

The specific growth rates of the eight microalgae strains tested using Screen 1.1 (Figure 2a) were well below the *μ*_max_ in TAP media (0.14 to 0.23 h^−1^). Consequently, a screen including micronutrients (Screen 2) was conducted to identify additional limiting nutrient factors.

For this purpose, the three best performing microalgae strains, *M. convolutum* (9-FW), *M. reisseri* (13) and *P. falcate* (4A-1) were chosen. They were tested in Screen 2 in triplicate using 4.2 mM NH_4_Cl and 10 mM KH_2_PO_4_ for strains *M. convolutum* (9-FW) and *M. reisseri* (13) and 30 mM NH_4_Cl and 10 mM KH_2_PO_4_ for strain *P. falcate* (4A-1) as the starting point. These Screen 1 formulations yielded specific growth rates of 0.061 h^−1^, 0.083 h^−1^ and 0.059 h^−1^ for strains *M. convolutum* (9-FW), *M. reisseri* (13) and *P. falcate* (4A-1), respectively. The best condition identified in Screen 1 forms the centre point (CP) of Screen 2 (‘Material and methods’) and acts as the baseline reference for Screen 2.

Figure 2b shows a photographic overview of Screen 2 at the 72-h endpoint. The two key points to note are that the 180 condition Screen 2 *Box-Behnken* analysis yielded a broad range of different growth rates and final biomass densities (light to dark green) and that some of the conditions tested yielded culture densities much closer to those obtained with the TAP control than was achieved in Screen 1.1. Based on these results, all of the curves were individually subjected to the curve fit quality control analyses (see Figure 6) and specific growth rates were then determined for each of the 180 Screen 2 conditions tested. Figure 3 summarises this information as a radial plot of the average (triplicate) specific growth rates (h^−1^) obtained for the three strains (*M. convolutum* (9-FW) (black), *M. reisseri* (13) (blue) and *P. falcate* (4A-1) (red)) for each of these 180 conditions. The first point of note is that the average of the Screen 2 specific growth rates is the same as the best conditions obtained in Screen 1. This is because 20 of the Screen 2 conditions are identical with the best conditions from Screen 1 and are used as the midpoint controls (centre points). Closer analysis also shows that several conditions yielded growth rate values significantly above (0.108 h^−1^, 0.138 h^−1^, 0.119 h^−1^ for *M. convolutum* (9-FW), *M. reisseri* (13) and *P. falcate* (4A-1), respectively) this midpoint (0.084 h^−1^, 0.12 h^−1^, 0.078 h^−1^ for *M. convolutum* (9-FW), *M. reisseri* (13) and *P. falcate* (4A-1), respectively) and much closer to the positive TAP controls (0.193 h^−1^, 0.2 h^−1^, 0.175 h^−1^ for *M. convolutum* (9-FW), *M. reisseri* (13) and *P. falcate* (4A-1), respectively). The best conditions obtained for the three strains are summarised in Table 3.

**Table 3:** **Specific growth rates and best medium composition in Screen 2**

**Strain**	**Highest** ***μ***_**max**_ **[h** ^**−1**^ **]**	**Centre point** ***μ***_**max**_ **[h** ^**−1**^ **]**	**TAP control** ***μ***_**max**_ **[h** ^**−1**^ **]**	**Improvement over Screen 1.1 (%)**	**Medium with highest** ***μ***_**max**_	**Element variation in best media**
*M. convolutum* (9-FW)	0.108 ± 0.010	0.080 ± 0.008	0.191 ± 0.005	44	177	Ca (+1), Mn (+1), B (−1), V (−1)
*M. reisseri* (13)	0.138 ± 0.016	0.118 ± 0.004	0.200 ± 0.021	40	175	B (+1), Fe (+1), Si (+1), Cu (−1)
*P. falcate* (4A-1)	0.119 ± 0.017	0.077 ± 0.008	0.175 ± 0.012	50	79	Ca (+1), Fe (+1), Zn (+1), Se (+1)

Algal strains were cultivated in triplicates.

### Main and interacting element effects

Next, the highest specific growth rate values from the Screen 2 trials were subjected to *Main effects* (*p* value) analysis using Minitab (Figure 4a). For a given element (for example, Ca), this analysis involved as follows: 1) The clustering of all conditions with low calcium (−1), medium calcium (0) and high calcium (1) concentrations; and 2) Calculating the average specific growth rate and standard deviation of each of these −1, 0 and 1 clusters and determining whether there is a significant difference between them (*p* ≤ 0.05). In the case of calcium (Figure 4a), it can be seen that at higher concentrations (1), higher growth rates are achieved than at lower concentrations (−1). A similar analysis was conducted for Ca, Mg, Fe, Cu, Mn, B, V, Si, Zn and Se (Figure 4a) for all eight species. The statistical analysis showed (Figure 4b) that of the eight strains tested, calcium had a significant effect for three strains as did Fe and Zn, while Mg affected two of eight (strains *M. convolutum* (9-FW) and *M. reisseri* (13)). The system is also designed to analyse pairwise interactions using Minitab (Figure 4c). Such analysis can identify a number of potential species/nutrient mix specific effects (for example, Mg-Zn in *M. convolutum* (9_FW)) or interactions common to more than one species (for example, Ca-Mg for *P. falcate* (4A-1) and *M. reisseri* (13)).

### Performance improvements through nutrient optimisation

The statistical *Main* and *Interaction Effects* analyses of Screen 2 as well as the analyses of its individual media compositions resulting in improved growth determined that Ca, Mg, Zn and Mn were for some species being supplied at insufficient levels. To establish proof of principle of the utility of the nutrient screen and to test whether the adjustment of Ca and Mg (highest and most frequent significance) in Screen 1 would result in significant improvement in the observed maximum specific growth rates, Screen 1.2 was tested. It differed from Screen 1.1 in that it used a fourfold increase of Ca (raised from 0.213 to 0.85 mM) and Mg (raised from 0.375 to 1.5 mM as the new baseline concentration (Table 2). The same eight microalgae strains tested in Screen 1.1 were then tested in Screen 1.2.

All strains showed better photoautotrophic growth performance in Screen 1.2 than in Screen 1.1 as shown in (Figure 5).

As expected, photoautotrophic growth rates were still below the photoheterotrophic rates (TAP controls). Most microalgae strains preferred media containing 3.8 mM NH_4_NO_3_ as the nitrogen source, and this also yielded the highest OD_750_ after 72 h of cultivation (endpoint). Most strains were also able to grow in all tested nitrogen sources supplied in the Screen 1*.*2 and growth trends seemed to be directly correlated to nitrogen and phosphate concentration levels (Figure 3b). The preferred phosphate concentrations varied between 2 and 10 mM, perhaps reflecting the capacity of the different algal strains to store phosphate intracellularly. The majority of strains, however, required only 2 mM phosphate (KH_2_PO_4_) to achieve high endpoint OD_750_ values.

In Screen 1*.*2, NH_4_^+^ salts are observed to be the most readily accessible and energy efficient (requiring less energy for assimilation) source of nitrogen [16] although at high concentration, it can cause toxicity [17-19] and variation in pH. This may also be due to pre-adaptation of the algae to TAP media (maintenance media of the strains), which contains NH_4_^+^ as nitrogen source. However, the two strains *C. pyrenoidosa* (22) and *P. falcate* (4A-1) showed good growth in NH_4_^+^ but grew best in urea ((NH_2_)_2_CO) despite pre-adaptation to NH_4_Cl from TAP media. Strains *M. inermum* (18-1), *A. gracilis* (18-2), *C. sorokiniana* (21) and *M. reisseri* (13) showed the highest specific growth rates with ammonium nitrate as the N source, perhaps because the ammonium concentration is low enough to avoid toxicity, but the nitrate still provides excess capacity on ammonium depletion.

### Ca and Mg

The Screen 1*.*2 experiments demonstrated that reformulation of calcium and magnesium (based on Screen 2 results) can increase the performance of the microalgae in both growth rate and endpoint OD_750_. In the future, re-optimisation of nutrients such as zinc and manganese, bioprospecting of new strains and pre-adaptation of the current microalgae strains with the improved media followed by re-screening and re-evaluation of physical cultivation parameters (optimisation of CO_2_ level, temperature and light intensity) are some approaches that may generate further improvements in growth rate and biomass accumulation that maximises photoautotrophic growth potential.

The significance of calcium and magnesium for photoautotrophic microalgal growth is interesting, as they are important elements in the microalgal photosynthetic apparatus. Calcium is part of the water oxidizing complex [3] of photosystem II and an important element in the CO_2_ fixation process in the Calvin-Benson-Bassham cycle (CBB cycle, reductive pentose phosphate cycle) which is the metabolic pathway that connects photosynthetic energy production to the conversion of atmospheric CO_2_ into organic compounds [20]. Calcium may also be involved in ion transport [21], which is important in nutrient uptake, and physico-chemical processes such as buffering, precipitation or interactions with toxic components. Magnesium, on the other hand, accounts for 2.7% of the molecular weight of chlorophyll and is necessary for chloroplast structure [22] involving the formation of grana from the thylakoid stacks [23]. Magnesium is also involved in chloroplast synthesis and in microalgal metabolism (it occurs in many cellular enzymes such as RNA polymerase, ATPases, protein kinases, phosphatases, glutathione synthase and carboxylases). Appropriate formulation of calcium and magnesium concentrations are therefore important for core metabolic and photosynthetic processes in the microalgal cells and can influence growth performance. From the literature, it was not predicted that these would be the most crucial nutrients to be found. Clearly, much more targeted experiments are required to identify the nature of these nutrient effects and interactions.

### Screen performance

The system defined optimal search spaces of nutrient concentrations and combinations and identified potential nutrient interactions and nutrient toxicity. The specific growth rates obtained are in broad agreement with the larger scale cultivation values reported in the literature [24]. The system was validated in terms of well-to-well variability (repeatability test by using Cronbach’s alpha analysis of 92% to 96% accuracy), run-to-run variability (reproducibility test standard deviation of 2.1% to 16.9% accuracy) and with respect to potential artefacts such as edge effects through evaporation. The data validity was found to be within the satisfactory error range. The best nutrients blend for each strain, and the corresponding nutrient main effects and interactions identified provided solid basis for further full factorial refinement of additional variables such as light quality and intensity and CO_2_ concentration in scaled up systems better suited for this purpose.

### Future optimisation

#### Lag phase reduction

In suitable media conditions, the lag phase lasted approximately 6 to 9 h prior to log growth phase. The lag phase length is dependent on species, the metabolic state of the cells as well as photoinhibition, and could potentially be reduced by using improved pre-culture conditions for individual species.

#### Nutrient range optimisation

An important consideration is the nutrient concentration range covered between the −1 and +1 conditions of the *Box-Behnken* analysis. Initially, the default condition was to use half (−1) or twice (+1) the average concentration. However, unlike the macronutrient case, it is possible that optimal ranges of micronutrients can vary by orders of magnitude, so that much wider ranges should be investigated in future designs. Future rounds of screening with wider micronutrient ranges may be required to uncover these ranges for specific situations. However, based on Screens 1.2 and 2 as well as main effects and pairwise interaction analysis, the optimisation space can be greatly reduced and the analysis of these variables ranked according to their relative importance.

## Conclusions

While a range of statically optimised processes have been reported [25-27], these have only been tested using small experimental arrays (for example, approximately 16 to 20 experiments). The new miniaturised and automated 1,728 multiwell *Screen* format presented here has enabled the analysis of a large multidimensional space (Screen 1: N and P full factorial; Screen 2: ten elements at three concentrations) for nutrient sufficiency (and minimisation of toxic effects) at three levels. The broad statistical space sampled (3^10^ = 59,049 full factorial conditions) was compressed 328-fold and successfully analysed using an incomplete factorial *Box-Behnken* design and required only 246 trials per cell line. This analytical method is therefore a powerful tool that can provide greatly improved data matrices over those reported to date. Furthermore, it yielded the *main effects* (for example, main nutrient effects on growth), identified *pairwise nutrient interactions* (for example, Ca-Mg) and provided the basis for a smaller targeted full factorial screen-set to assist with optimisation of algae production processes for scale up in terms of energy balance and economic return. The further integration of photographic recording, FACS analysis and FTIR screening opens up the opportunity to extend analyses to factors affecting cell aggregation, cell division and metabolic pathways. The rates obtained compare favourably with those reported in the literature [24]. This can be explained by near optimal nutrient provision under conditions in which light and CO_2_ are not limiting low cell concentrations and thin cultures [28].

## Material and methods

### High-throughput screen design

The nutrient screen was designed to provide a miniaturised, automated, high-throughput platform for rapid low-cost optimisation of nutrient conditions. The 96-well microwell plate format was chosen as a basis for the design as it provides future flexibility to expand sample scaling (for example, 384-, 96-, 48-, 24-, 12- and 6-well) as well as array scalability (for example, from the current eighteen 96-well plates = 1,728 wells) while achieving acceptable errors associated with miniaturisation.

Optical density (OD_750_), which is a measure of light scattering, was used as a proxy for biomass and to determine microalgal growth rates [29]. It was chosen as it is a standard measure of growth kinetics and is highly correlated with biomass yield. Furthermore, it provides the required precision and accuracy for this broad screen and is cheap, simple and suited for automation. The use of OD_750_ eliminates the effects of varying chlorophyll content of the cells. Although OD_750_ solely measures light scattering and so does not differentiate between algae, bacterial or fungal biomass, detritic compounds or algal exudates, the use of axenic algae cell cultures eliminated most of these complications. Subsequent further precision testing of high-performance conditions identified during the screen can be conducted to deliver higher precision if required. However, as OD750 is based on light scattering, it is influenced by cell size. For the purposes of this broad screen, it is therefore necessary to assume that cell size does not vary significantly over the duration of the experiment. This is clearly an approximation. However, the screens were species specific (measuring intra-species variance related to nutrient effects) and the average cell size of most algae species do not vary more than two- to threefold in diameter throughout the growth cycle. Unless synchronised, this is the case for most populations containing a mixture of cell sizes [30]. Cultivation was conducted for 75 h or less to minimise evaporation effects. A second-generation system design could potentially incorporate additional checks for accuracy of biomass estimation.

### Automated media preparation

A Tecan robot (Freedom Evo 150, Tecan Group Ltd., Männedorf, Switzerland) (Figure 1a) equipped with a liquid handling arm (1) was used to accurately dispense the nutrient screen matrix stock solutions into 1,728 wells. The liquid handling arm (1) dispenses the stock solutions from a set of 100-mL troughs (2) into eighteen 96-well plates placed on two platforms (3) to generate the nutrient blend matrices required for growth trials. A large trough (4) located between the troughs (2) and platform (3) was used to wash the tips between the dispensing steps of different nutrients. The dispensed nutrient plates were then gamma sterilised at a dose of 2 k-Gy. Microwell plates containing the media were wrapped in cling film and were stored at −20°C until used. A filter sterilised vitamins B_1_ and B_12_ solution (Acrodisc 0.22-μm filter) was added to the gamma sterilised media together with the microalgae inoculum (see Additional file 1: Figure S2, S3 and Additional file 1: Table S2).

### Automated growth chamber

#### System layout

A second Tecan system (Tecan Freedom EVO 150 robotic workstation, Tecan Group Ltd., Männedorf, Switzerland) was configured and further developed into an automated microalgae growth chamber (Figure 1b). Specifically, it was fitted with three orbital shakers (IKA KS 130 Control microwell plate shakers, IKA Werke GmbH & Co., KG, Staufen, Germany), each holding six 96-well microwell plates (5), enabling the use of a total of eighteen 96-well plates (1,728 samples). The system was operated at room temperature and during the experiment remained within a range of 23°C ± 0.5°C.

#### Illumination

Controlled top illumination (Figure 1b (6)) and bottom illumination (7) have been integrated into the system, with capacity for both continuous illumination and day/night cycling. The top illumination system was designed to closely match the visible part of the solar spectrum. It consists of alternating fluorescent lights (12 Cool white Phillips PL-L55W/840 Cool White, Philips International B.V., Amsterdam, Netherland, and 11 Philips PL-L55W/830 Warm White lights, Phillips International B.V., Amsterdam, Netherland). The fluorescent light sources extend beyond the whole cultivation area and were positioned over approximately 1.5 m above the microwell plates, to ensure even illumination. Uniformity of illumination across the full cultivation area was confirmed through detailed light meter measurements and achieved a maximum light intensity of 450 μmol photons m^−2^ s^−1^ at the microwell plate level.

Below the microwell plates, a customised diode array lighting system was also fitted (see Figure 1b insert). This illumination system positioned one light-emitting diode (SMD 3020, Epistar, Hsinchu City, Taiwan) below each well of each 96-well plate (LEDs are rated to +/−5%). The maximum illumination intensity is approximately 3,000 μmol photons m^−2^ s^−1^ and can be adjusted between 0% and 100% of maximum intensity in 1% increments. This ability to vary light intensity enables ‘dynamic’ day/night cycling. Programs coded in Arduino© (Arduino SA) provide the ability to run: (1) A fixed light cycle, (2) A day/night cycle with light flux changing at manually set time increments (for example, 5% every 30 min to a maximum or minimum level) to simulate outdoor solar conditions and (3) a rapid flashing light cycle to simulate mixing of cells in photobioreactors (maximum cycle speed is 10 ms^−1^). The top and bottom illumination systems can be used individually or in combination.

#### CO_2_ control

The growth chamber was also fitted with an atmospheric CO_2_ control system (Get Red-y 5 system, Voegtlin Instruments AG, Aesch, Switzerland). Specifically, two thermal mass flow controllers (Red-Y Smart Controllers, Voegtlin Instruments AG, Aesch, Switzerland) were fitted to regulate the mass flow of air and CO_2_ into the chamber based on the measured CO_2_ concentration. The CO_2_ concentration was measured using the CO_2_ probe (CARBOCAP® GMT 220 CO_2_ probe, Vaisala, Oyj, Finland) shown in Figure 1b (8). To minimise the use of CO_2_ required to maintain a stable 1% enriched atmosphere, a specifically designed low wall mounting (9: dimensions: 110 cm × 45 cm × 13 cm) was fitted around the shakers. The volume within it (approximately 65 L) is approximately 11 times less than the total volume of the entire Tecan enclosure (dimensions 115 cm × 130 cm × 50 cm) and, as it does not have a top, does not interfere with the light path from the top lights. A stable 1% ± 0.3% CO_2_-enriched atmosphere could therefore be maintained much more precisely and with a reduced CO_2_ requirement by flooding the 1% CO_2_ mix into the bottom of the enclosure via a looping perforated tube system.

#### Time course assays

A robotic manipulator arm (10 - Tecan, ROMA) was fitted to transfer the plates to a plate reader after removal of the lid (11 - Tecan Infinite M200 PRO, Tecan Group Ltd., Männedorf, Switzerland) to measure optical density at defined intervals (typically every 3 h).

### Algae growth media variations for the screening

The ‘midpoint’ and elemental screen range of the screen was based on an extensive literature search and the average values obtained. In total, 11 different fresh water media (TAP medium [31], HSM medium [32], Johnson medium [33], Bristol medium [34], *Botryococcus* medium [35], *Spirulina* medium [35], M4N medium [36], Modified Bold 3 N [35], Del Río medium [37], BG11-1 medium [38] and Modified BG11 medium [39]) were analysed, and their elemental compositions are compared in Additional file 1: Table S3. NaNO_3_ and NH_4_Cl were found to be the most common nitrogen sources. In this screen, urea ((NH_2_)_2_CO), a common and cheap fertiliser, and ammonium nitrate (NH_4_NO_3_), which provides an opportunity for microalgae to dynamically switch N sources during growth, were also tested.

For microelements, the Hutner’s trace formulation [40] was modified by the inclusion of selenium, vanadium and silicon. Other elements have been included because many elements are not essential but beneficial for growth and to make the screening systems applicable to a broad variety of microalgae strains, such as diatoms. The average nutrient concentration based on these 11 media was used as the average values for Screen 1 and the initial mid-value for the Screen 2 system. It was noted that average concentration values derived from the literature search analysis may not be optimal but provided a sensible starting point for optimisation. Solubility constants of each element were examined to ensure that the formulation did not induce precipitation.

Careful formulation of the microelements was crucial to produce accurate and sensible information from the nutrient screen systems for application to the larger scale systems such as bioreactor and open pond systems. Selenium (0.1 μM) [14], vanadium (0.009 μM) [38], silicon (273 μM) [38], vitamin B_1_ (52 μM) [15,41] and vitamin B_12_ (0.1 μM) [15,42] were used as a baseline of both screens in addition to the Hutner’s trace elements [40] and concentrations (Table 1) used for TAP media [31]. In addition, 0.5373 mM Na_2_-EDTA, pH 8.0 (chelating agent) and 100 mM Tris-HCl (pH 7.4) buffer are added to the formulation (concentrations derived from range finding experiments, data not shown). Extensive preliminary trials were conducted to monitor optical density changes of the screen media over an experimental run period to ensure that no salt precipitation occur that could contribute to increased measured OD. Given this and to maximise the efficiency of statistical design, blank wells were not included in the runs. This is however optional.

To optimise the efficiency of the screen statistically (that is, to maximise the multidimensional search space and minimise sample number), the screen was configured into a two stages process (Screen 1 and Screen 2).

### Screen 1 - N and P optimisation

Screen 1 was designed to identify the best N type and concentration tested (Figure 2), and these are based upon the average literature values (Additional file 1: Table S3) and the concentration ranges listed in Table 1. The rationale for this approach is that different algae have different N preferences and that the effects of N and P are so important that without their initial optimisation, the statistical influences of the other elements on algae growth will be masked. For example, ammonium requiring algae would show very low growth in nitrate based media.

The nitrogen (N) source concentration was adjusted to account for the number of N atoms in the source (for example, NaNO_3_ = 1, NH_4_NO_3_ = 2). A chelating agent (0.5373 mM Na_2_-EDTA, pH 8.0) and a buffer (100 mM Tris-HCl, pH 7.4) were added to the formulation (concentrations derived from range finding experiments - data not shown). It is recognised that such high levels of EDTA and Tris-HCl would not likely be suitable for subsequent scale up cultivations; however, they are required here to ensure pH stability and to prevent precipitation in a miniaturised system that cannot be controlled in an automated fashion as in scaled up photobioreactors.

The full factorial design of Screen 1 investigates the effect of the four different nitrogen sources and one phosphorous source at five and three concentration levels, respectively (Table 1), for each algal strain in the test. In total, Screen 1 consists of 60 different photoautotrophic conditions and 3 positive photoheterotrophic controls (TAP media).

*Nutrient Screen 2* uses the best N and P conditions from Screen 1 and is based on the statistical incomplete factorial *Box-Behnken* design. It is designed to measure the effects of Ca, Mg, Fe, Mn, Cu, Zn, B, Se, V and Si on microalgal growth performance. The elements were tested at three concentration levels coded as −1 (low), 0 (middle) and +1 (high). Other nutrients were supplied at constant concentrations. These consisted of CoCl_2_, (NH_4_)_6_Mo_7_O_24_, Na-EDTA (pH 8), Tris-HCl (pH 7.4) and vitamins B_1_ and B_12_ (Table 1) which excludes them from being tested variables in the current screen configuration. These elements, though not a complete set of nutrients at this stage, were considered to be the most critical for initial testing of a broad range of species. The low and high concentration levels for each of the nutrient elements were set as a twofold difference from the middle concentration (Table 1). The *Box-Behnken* experimental design allows the observation of primary effects and nutrient interaction effects on microalgal growth to be determined and presented via response surface analysis [11]. Minitab 15 software (Minitab Inc., State College, PA, USA) was used to design the experiment and generated 180 different media formulations (experiments) (Additional file 1: Table S4 ).

The three-level second-order response surface model for *m* factors (x_1_,…, x_*m*_) in *n* runs is described by Equation 1 [43].

1 $$ y=X\beta +\varepsilon $$

*y* = the *n* × 1 response vector

*n* = number of runs (equals number of concentrations tested)

*X* = *n* × *p* model matrix with *n* 1 × *p* row vectors

*x* = (1, *x*_1_,…, *x*_m_, *x*_1_*x*_2_,…, *x*_*m*−1_*x*_*m*_, *x*_1_^2^,…, *x*_*m*_^2^)

*m =* number of factors (here 10)

*x*_*m*_ *=* growth rate of factor *m*

*β* = *p* × 1 vector of parameters (to be estimated)

*ε* = *n* × 1 vector of errors (with zero mean and covariance matrix *I*_*n*_*σ*^2^)

### Screen format

A total of 24 species (or 8 species in triplicates) can be analysed in a single Screen 1 run, and a total of 9 species (or 3 species in triplicates) in 180 conditions can be analysed in Screen 2. All nutrient elements were prepared as individual stocks. Both screens include a triplicate photoheterotrophic/mixotrophic growth condition controls in TAP media to compare between runs of the same strain (quality control) as well as to photoautotrophic growth conditions. Microalgal growth rates in media containing acetate as additional carbon source (TAP controls) are expected to be higher than rates in photoautotrophic growth conditions using CO_2_ as sole carbon source.

### Growth rate determination

Assuming that the specific growth rate *μ* (h^−1^) represents the average growth rate of all cells present in the culture, it defines the fraction of increase in biomass over a unit of time and is proportional to the biomass of the cells during exponential growth phase (Equation 2). OD_750_ was used as the basis for maximum specific growth rates *μ*_max_ determination of each condition. These rates were used to compare different conditions within the nutrient screens for each algae strain. In general, batch culture growth phases can be divided into lag, exponential, linear and stationary phase with *μ*_max_ occurring in exponential phase.

2 $$ \mu =\left(\mathbf{ln}\;\mathbf{O}{\mathbf{D}}_{750\left(\mathbf{t}2\right)}\hbox{--} \mathbf{ln}\;\mathbf{O}{\mathbf{D}}_{750\left(\mathbf{t}1\right)}\right)\;/\;\left({t}_2-{t}_1\right) $$

*μ* = specific growth rate

OD_750(t1)_ = OD_750_ at time

OD_750(t2)_ = OD_750_ at time

*t*_1_ = time 1 (h)

*t*_2_ = time 2 (h)

High-throughput data processing requires a form of curve fitting that appropriately compensates for irregularities, such as circadian rhythm or scattering effects, to ensure a high comparability of different growth conditions (see Equation 3). Under optimal growth conditions, the microalgae growth curve from lag phase to stationary phase can be usefully described by a sigmoidal curve. Non-linear regression was used to normalise (curve fit) the recorded 3-h OD_750_ data points to a simple sigmoidal model (variable sigmoidal curve fit (GraphPad Prism, GraphPad Prism Inc., La Jolla, USA)) (Equation 3). The sigmoidal curve fit approach was selected because it describes the usual physiological behaviour of the system where reasonable growth occurs. In non-ideal growth conditions where specific growth rates are low, a sigmoidal fit cannot be achieved (for example, linear growth with no plateau). Strong circadian rhythms in some algae can also interfere with curve fitting (Figure 6). Under these conditions, the growth rates were excluded from the screen.

3 $$ \mathrm{Y}=\mathbf{k}{\mathbf{t}}_{\mathbf{o}}+\left(\mathbf{k}{\mathbf{t}}_{\mathbf{n}}\mathbf{\hbox{--}}\mathbf{k}{\mathbf{t}}_{\mathbf{o}}\right)/\left(\mathbf{1}+\mathbf{1}{\mathbf{0}}^{\left(\mathbf{log}\ {t}_{\frac{1}{2}}\mathbf{\hbox{--}}t\right)*\mathbf{Hill}\;\mathbf{slope}}\right) $$

*Y* = normalised OD_750_ data xpoint,

kt_o_ = raw OD_750_ at time 0,

kt_n_ = raw OD_750_ at time n,

log *t*_½_ = log_10_ of time when the OD_750_ is between *t*_0_ and *t*_n_,

Hill slope = the steepness of the curve at t_½_.

Specific growth rates were then determined using sigmoidal fitted 3-h OD_750_ data points and calculating the slope of two consecutive data points (Equation 2). The highest slope value represents *μ*_max_ of a condition. Good growth conditions were selected by comparing all *μ*_max_ values for each strain.

The quality of the fit was assessed using *R-*square (where a value more than 0.85 was chosen to indicate good quality) and *absolute sum of squares* (value less than 0.1 was chosen to indicate good quality) (Figure 6). A data cutoff limit based on the *R-*square (*R*^*2*^) value of the normalised growth plots was applied. This was designed to screen and remove fitted growth curves with *R*^*2*^ smaller than 0.85. Growth curves that can be fitted accurately to the regression model have smaller sum of square regression (SS_reg_) than sum of square total (SS_tot_). The curve fitting process first generates a number of possible curve fits to the raw data and then identifies the model yielding the highest *R*^*2*^ and the least sum of square. In the situation in which only limited data can be fitted or the chosen model is too complicated, the *Not Converged* or *Ambiguous* remarks respectively are generated by the GraphPad Prism software [44].

Although the screen was validated through triplicate runs, each screen is designed to be conducted without replicates to broaden the screen by maximising the number of conditions and algae strains assayed per run. The screen is not intended to be highly analytically precise but to identify optimal regions of nutrient search space which can be analysed more precisely using conventional assays, while excluding the vast majority of conditions. Validation of well-to-well (repeatability test) and run-to-run reliability (reproducibility test) indicated excellent internal data consistency between replicate experiments (see Additional file 1: Figures S4, S5 and S6), in particular for high growth rate samples.

### Statistical analysis on microalgal growth rate using response surface method

The *Main* and *Interaction Effects* analyses (*response surface method* (RSM)) were used to identify specific effects and statistical interactions between the nutrients as well as to determine the significance of nutrients that can improve microalgal growth.

### Main and Interaction Effects analysis

The *Main Effects* analysis identifies the statistical significance of individual nutrients on the microalgae growth rate (Figure 4). The *Interaction Effects* analysis determines significant statistical interactions between multiple nutrient factors and their effects on microalgal growth rate. When the *Main* and *Interaction Effects* exhibited significance (*p* ≤ 0.05), the nutrient factor involved in these cases should be fine-tuned for growth performance improvement. The analysis is based on the average value of the growth rate of specific nutrient concentration level (−1, 0 and 1) in the changing background of 180 experiments conducted within the *Box-Behnken* matrix. In principle, important conditions will significantly influence the relative growth rates within these changing backgrounds.

The *Interaction Effects* analysis determines the synergistic or antagonistic effects of two nutrient elements on microalgal growth rates. Nutrient elements that exhibited statistical significant interaction effects could be subsequently evaluated or optimised on a rational basis to increase the microalgae growth rate. The analysis was based on the average value of the growth rate of specific nutrient concentration levels (−1, 0 and 1) from 180 experiments (Additional file 1: Table S4).

### Algae strains and culture conditions

The microalgae strains *M. inermum* (18-1), *A. gracilis* (18-2), *R. complanata* (SF-150), *C. sorokiniana* (21), *M. convolutum* (*9-FW*), *C. pyrenoidosa* (22), *M. reisseri* (13), *P. falcate* (4A-1) were isolated in the vicinity of Brisbane, Queensland, Australia. Identification consisted of morphological investigation (Olympus BX42 and Nikon Ti-U, × 200 and × 400 magnification) and molecular classification by rDNA analysis (see Additional file 1: Figure S1 and Table S3). The amplification of 18S rDNA and its sequencing was outsourced to the Australian Genome Research Facility (AGRF). Sequences were aligned using nucleotide BLAST (NCBI, http://blast.ncbi.nlm.nih.gov/Blast.cgi) against the ‘nucleotide collection (nr/nt)’ database.

Microalgae cells from agar plates (TAP + 0.3% yeast extract + 1.5% agar) grown at 23°C in 50 μmol photons m^−2^ s^−1^ were used to build up inoculation cultures grown in 150-mL flasks with TAP medium [31] (23°C, 120 μmol photons m^−2^ s^−1^) on an orbital shaker (approximately 120 rpm). Algae strains that did not tolerate acetate were grown in tris phosphate (TP) media only. Algal strains originating from brackish water were supplemented with 250 mM NaCl. Cell densities were determined using optical density measurements at 750 nm (OD_750_) using a microwell plate reader (Infinite M200 PRO, Tecan Group Ltd., Männedorf, Switzerland). Algal cells during log phase growth were collected by centrifugation (500 *g*, 10 min, 25°C using Hettich Zentrifugen Universal 320R, Hettich Instrument Inc., Beverly, USA) and washed once before resuspending in 100 mM TRIS buffer (pH 7.4). The cells were inoculated into sterile 96-well plates, each well having an individual media composition using a starting OD_750_ of 0.1 using the microwell plate reader. All algae strains were grown in 150 μL in 96-well plates (5-mm culture depth) on an orbital shaker (580 rpm) under continuous light using top illumination (120 μmol photons m^−2^ s^−1^) at 23°C ± 0.5°C and 1% CO_2_ atmosphere (±0.3% CO_2_).

## Additional file

Additional file 1:
**Microalgae characterisation, microwell sterilization techniques, cultivation media comparison, Screen 2 media key and statistical screening background.** The additional file contains: Microalgal identification data for the strains used based on molecular (Additional Table 1) and morphological (Additional Figure 1) classification by rDNA analysis; Optimisation data of gamma irradiation levels for nutrient media sterilization (Additional Figure 2) and its effect on vitamin B_1_ and B_12_ stability (Additional Figure 3) as well as on *Chlamydomonas reinhardtii* growth (Additional Table 2); A list of average macronutrient concentrations in common media (Additional Table 3); Individual Screen 2 media conditions; Notes on statistical variability, *Cronbach Alpha* analysis for Screen 1.2 (Additional Figure 4) and Screen 2 (Additional Figure 5); Analysis of the run-to-run reproducibility of the highest growth rate values obtained in Screen 2 (Additional Figure 6).

## Abbreviations

ATPase: adenosinetriphosphatase
CBB cycle: Calvin-Benson-Bassham cycle
CP: centre point
FACS: fluorescence-activated cell sorting
FTIR: Fourier transform infrared spectroscopy
OD_750_: optical density at 750 nm
TAP: Tris-acetate-phosphate medium
*μ*_max_: maximum specific growth rate
