# Supplementary material for: Automated nutrient screening system enables high-throughput optimisation of microalgae production conditions
Source: Biotechnol Biofuels. 2015 Apr 11;8:65. doi: 10.1186/s13068-015-0238-7 (PMC4432509; doi:10.1186/s13068-015-0238-7)
Supplement: Additional file 1: — Microalgae characterisation, microwell sterilization techniques, cultivation media comparison, Screen 2 media key and statistical screening background. The additional file contains: Microalgal identification data for the strains used based on molecular (Additional Table 1) and morphological (Additional Figure 1) classification by rDNA analysis; Optimisation data of gamma irradiation levels for nutrient media sterilization (Additional Figure 2) and its effect on vitamin B1 and B12 stability (Additional Figure 3) as well as on Chlamydomonas reinhardtii growth (Additional Table 2); A list of average macronutrient concentrations in common media (Additional Table 3); Individual Screen 2 media conditions; Notes on statistical variability, Cronbach Alpha analysis for Screen 1.2 (Additional Figure 4) and Screen 2 (Additional Figure 5); Analysis of the run-to-run reproducibility of the highest growth rate values obtained in Screen 2 (Additional Figure 6). [file 13068_2015_238_MOESM1_ESM.doc]

**Additional files:**

**Microalgae identification**

Microalgal identification was based on molecular (Additional Table 1) and morphological (Additional Figure 1) classification by rDNA analysis.

**Additional Table 1: Molecular classification of the above algae was based on rDNA analysis. 18S ribosomal DNA analysis was performed. The amplification of 18S rDNA and its sequencing was outsourced to the Australian Genome Research Facility (AGRF). Sequences were aligned using nucleotide BLAST NCBI, (**[**http://blast.ncbi.nlm.nih.gov/Blast.cgi**](http://blast.ncbi.nlm.nih.gov/Blast.cgi)**) against the ‘nucleotide collection (nr/nt)’ database.**

| Strain | rDNA Sequence |
| --- | --- |
| *Micractinium inermum*  (18-1) | TAGATTGTACTCATTCCAATTACCAGACCTGAAAAGGCCCAGTATTGTTATTTATTGTCACTACCTCCCTGTGTCAGGATTGGGTAATTTGCGCGCCTGCTGCCTTCCTTGGATGTGGTAGCCGTTTCTCAGGCTCCCTCTCCGGAATCGAACCCTAATCCTCCGTCACCCGTTACCACCATGGTAGGCCTCTATCCTACCATCGAAAGTTGATAGGGCAGAAATTTGAATGAAACATCGCCGGCACAAGGCCATGCGATTCGTGAAGTTATCATGATTCACCGCGAGTCGGGCAGAGCCCGGTCGGCCTTTTATCTAATAAATACGTCCCTTCCAGAAGTCGGGATTTACGCACGTATTAGCTCTAGATTTACTACGGGTATCCGAGTAGTAGGTACCATCAAATAAACTATAACTGATTTAATGAGCCATTCGCAGTTTCACAGTATAAAGCAGTTTATACTTAGACATGCAGGCTAATCT |
| *Ankistrodesmus gracilis*  (18-2) | TTAGATTGTACTCATTCCATTACCAGACATTGAATGCCCGGTATTGTTATTTATTGTCACTACCTCCCCGTATCAGGATTGGGTAATTTGCGCGCCTGCTGCCTTCCTTGGATGTGGTAGCCGTTTCTCAGGCTCCCTCTCCGGAATCGAACCCTAATCCTCCGTCACCCGTTACCACCATGGTAGGCCTCTATCCTACCATCGAAAGTTGATAGGGCAGAAATTTGAATGAAACATCGCCAGCACAAGGCTATGCGATTCGTGAAGTTATCATGATTCACCGCGGGTCGGGCAGAGCCCGGTCGGCCTTTTATCTAATAAATACGTCCCTTCCAGAAGTCGGGATTTGCGCACGTATTAGCTCTAGAATTACTACGGTTATCCGTGTAGAGGTACCATCAAATAAACTATAACTGATTTAATGAGCCATTCGCAGTTTCACAGTATAAGCAGTTTATACTTAGACATGCATGGCTTAATCTT |
| *Rhombocystis complanata*  (SF-150) | TAGATTGTACTCATTCCAATTACCAGACATTGAATGCCCGGTATTGTTATTTATTGTCACTACCTCCCCCGTTATCAGGATTGGGTAATTTGCGCGCCTGCTGCCTTCCTTGGATGTGGTAGCCGTTTCTCAGGCTCCCTCTCCGGAATCGAACCCTAATCCTCCGTCACCCGTTACCACCATGGTAGGCCTCTATCCTACCATCGAAAGTTGATAGGGCAGAAATTTGAATGAAACATCGCCAGCACGAGGCTATGCGATTCGTGAAGTTATCATGATTCACCGCGGGTCGGGCAGAGCCCGGTCGGCCTTTTATCTAATAAATACGTCCCTTCCAGAAGTCGGGATTTACGCACGTATTAGCTCTAGAATTACTACGGTTATCCGTGTAGAGGTACCATCAAATAAACTATAACTGATTTAATGAGCCCATTCGCAGTTTCACAGTATAAGCAGTTTATACTTAGACATGCATGCGTAATCT |
| *Chlorella sorokiniana*  (21) | TTAGATTGTACTCATTCCAATTACCAGACCTGAAAAGGCCCAGTATTGTTATTTATTGTCACTACCTCCCTGTGTCAGGATTGGGTAATTTGCGCGCCTGCTGCCTTCCTTGGATGTGGTAGCCGTTTCTCAGGCTCCCTCTCCGGAATCGAACCCTAATCCTCCGTCACCCGTTACCACCATGGTAGGCCTCTATCCTACCATCGAAAGTTGATAGGGCAGAAATTTGAATGAAACATCGCCGGCGCAAGGCCATGCGATTCGTGAAGTTATCATGATTCACCGCGAGTCGGGCAGAGCCCGGTCGGCCTTTTATCTAATAAATACGTCCCTTCCAGAAGTCGGGATTTACGCACGTATTAGCTCTAGATTTACTACGGGTATCCGAGTAGTAGGTACCATCAAATAAACTATAACTGATTTAATGAGCCATTCGCAGTTTCACAGTATAAAGCAGTTTATACTTAGACATGCATGCTAATCTT |
| *Monoraphidium convolutum*  (*9-FW*) | TTAGATTGTACTCATTCCAATTACCAGACATTGAATGCCCGGTATTGTTATTTATTGTCACTACCTCCCCGTATCAGGATTGGGTAATTTGCGCGCCTGCTGCCTTCCTTGGATGTGGTAGCCGTTTCTCAGGCTCCCTCTCCGGAATCGAACCCTAATCCTCCGTCACCCGTTACCACCATGGTAGGCCTCTATCCTACCATCGAAAGTTGATAGGGCAGAAATTTGAATGAAACATCGCCAGCACGAGGCTATGCGATTCGTGAAGTTATCATGATTCACCGCGGGTCGGGCAGAGCCCGGTCGGCCTTTTATCTAATAAATACGTCCCTTCCAGAAGTCGGGATTTACGCACGTATTAGCTCTAGAATTACTACGGTTATCCGTGTAGAGGTACCATCAAATAAACTATAACTGATTTAATGAGCCATTCGCAGTTTCACAGTATAAGCAGTTTATACTTAGACATGCATGGCTTAATCT |
| *Chlorella pyrenoidosa*  (22) | GGTTTAGATTGTACTCATTCCAATTACCAGACCTGAAAAGGCCCAGTATTGTTATTTATTGTCACTACCTCCCTGTGTCAGGATTGGGTAATTTGCGCGCCTGCTGCCTTCCTTGGATGTGGTAGCCGTTTCTCAGGCTCCCTCTCCGGAATCGAACCCTAATCCTCCGTCACCCGTTACCACCATGGTAGGCCTCTATCCTACCATCGAAAGTTGATAGGGCAGAAATTTGAATGAAACATCGCCGGCACAAGGCCATGCGATTCGTGAAGTTATCATGATTCACCACGAGTCGGGCAGAGCCCGGTCGGCCTTTTATCTAATAAATACGTCCCTTCCAGAAGTCGGGATTTACGCACGTATTAGCTCTAGATTTACTACGGGTATCCGAGTAGTAGGTACCATCAAATAAACTATAACTGATTTAATGAGCCATTCGCAGTTTCACAGTATAAAGCAGTTTATACTTAGACATGCATGCTTAATCTT |
| *Micractinium reisseri*  (13) | TAGATTGTACTCATTCCAATTACCAGACCTGAAAAGGCCCAGTATTGTTATTTATTGTCACTACCTCCCTGTGTCAGGATTGGGTAATTTGCGCGCCTGCTGCCTTCCTTGGATGTGGTAGCCGTTTCTCAGGCTCCCTCTCCGGAATCGAACCCTAATCCTCCGTCACCCGTTACCACCATGGTAGGCCTCTATCCTACCATCGAAAGTTGATAGGGCAGAAATTTGAATGAAACATCGCCGGCACAAGGCCATGCGATTCGTGAAGTTATCATGATTCACCGCGAGTCGGGCAGAGCCCGGTCGGCCTTTTATCTAATAAATACGTCCCTTCCAGAAGTCGGGATTTACGCACGTATTAGCTCTAGATTTACTACGGGTATCCGAGTAGTAGGTACCATCAAATAAACTATAACTGATTTAATGAGCCATTCGCAGTTTCACAGTATAAAGCAGTTTATACTTAGACATGCAGGCTAATCT |
| *Podohedriella falcate*  (4A-1) | AGATTGTACTCATTCCATTACCAGACACTAAGTGCCCGGTATTGTTATTTATTGTCACTACCTCCCCGTATCAGGATTGGGTAATTTGCGCGCCTGCTGCCTTCCTTGGATGTGGTAGCCGTTTCTCAGGCTCCCTCTCCGGAATCGAACCCTAATCCTCCGTCACCCGTTACCACCATGGTAGGCCTCTATCCTACCATCGAAAGTTGATAGGGCAGAAATTTGAATGAAACATCGCCGGCACTAGGCCATGCGATTCGTGAGAGTTATCATGATTCACCGCGGGTCGAGCAAAGCTCGGTCGGCCTTTTATCTAATAAATACGTCCCTTCCAGAAGTCGGGATTTACGCATGTATTAGCTCTAGAATTACTACGGTTATCCATGTAGTAGGTACCATCAAATAAACTATAACTGATTTAATGAGCCATTCGCAGTTTCACAGTATATCAGTTTATACTTAGACATGCATGGCTTAATCT |

Additional Figure 1: Microscopic images of the microalgae strains screened. *(a) M. inermum*, (b) *A. gracilis*, (c) *R. complanata*, (d) *C. sorokiniana*, (e) *M. convolutum*, (f) *C. pyrenoidosa*, (g) *M. reisseri*  and (h) *P. falcate*. Scale bar: 50 µm. Microalgal identification consisted of morphological investigation (*Olympus BX42* and *Nikon Ti-U*, 200x and 400x magnification)


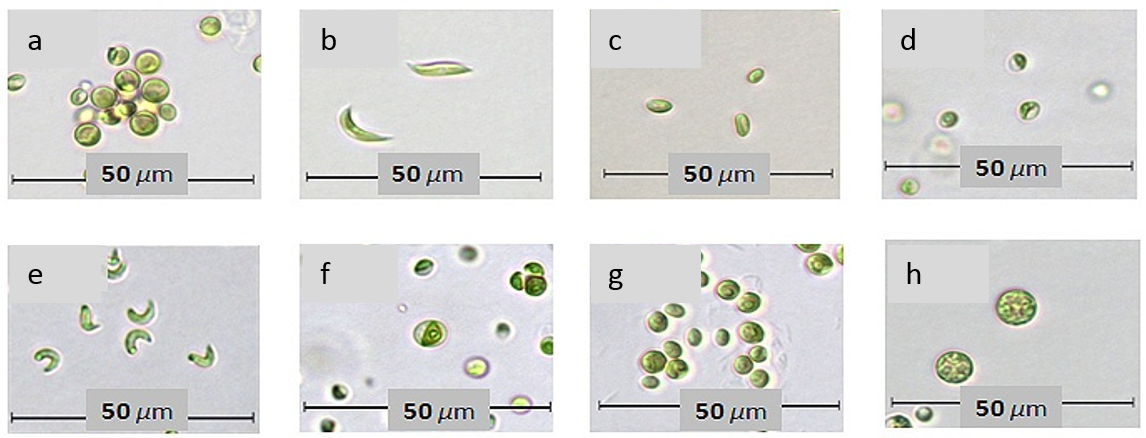


Additional Figure 2: Optimisation of gamma irradiation levels for nutrient media sterilisation. The gamma irradiation levels were tested in terms of their ability to sterilise LB medium inoculated with *E.coli* (inoculation density (OD_600 =_ 0.002. The samples were treated with 0.1, 0.5 0.8, 1, 2, 3 and 4.5 kGy, with non-gamma treated samples providing the positive control and LB media the negative control. Post-gamma irradiation, the flasks were incubated at 37^o^C for 8 days. The final OD_600_ were plotted and show that a dose of 2 kGy was sufficient to sterilise the inoculated medium.

**Additional Figure 3: Effect of gamma irradiation on vitamin B1 and B12 stability.** The UV-visible spectra of vitamin B1 and vitamin B12 before (0kGy) and after (2kGy) gamma sterilization show significant spectral shifts indicative of radiation-induced damage. Consequently these vitamins could not be gamma sterilised effectively and so were filter sterilised prior to addition to gamma sterilised cultivation media (See materials and methods).


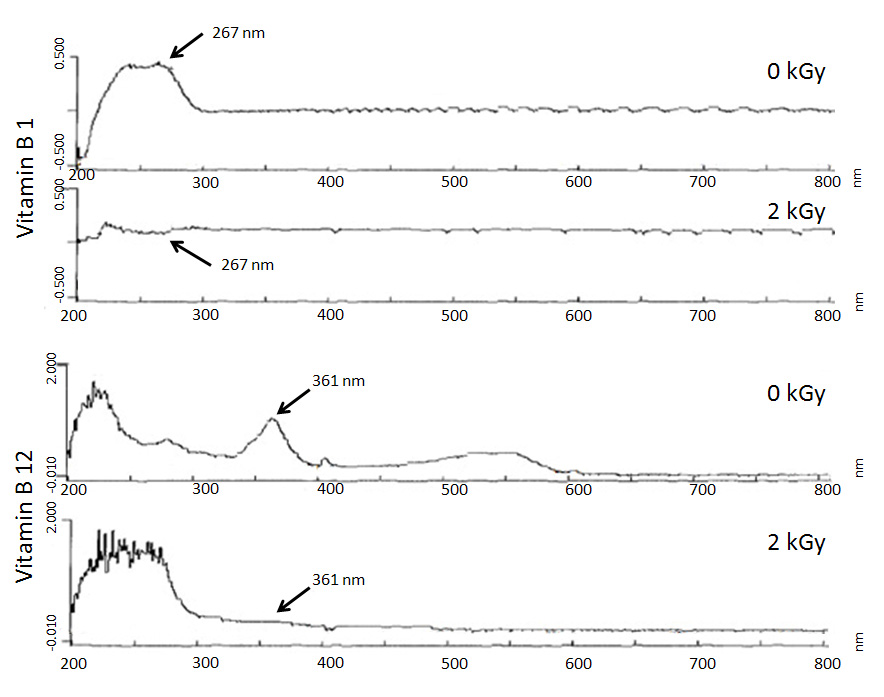


**Additional Table 2: t-test analysis shows that gamma sterilisation (2kGy) does not have a significant effect on the ability of nutrient screen 1 media to support *Chlamydomonas reinhardtii* growth.**

| Screen 1 media Treatment | N | Mean (OD_750_) | Standard Deviation | Standard Error Mean |
| --- | --- | --- | --- | --- |
| Gamma Sterilised | 120 | 0.315 | 0.240 | 0.022 |
| Non-gamma Sterilised | 120 | 0.324 | 0.270 | 0.025 |

Difference = µ (Gamma Sterilised) - μ (Non-Gamma Sterilised)

Estimate for difference: -0.0091

95% CI for difference: (-0.0741, 0.0559)

T-Test of difference = 0 (vs not =): T-Value = - 0.28, P-Value = 0.783 DF = 238

Both use Pooled StDev = 0.2557

**Additional Table 3:** Average macronutrient concentrations used in common media (see materials and methods).

| **Nutrient** | **TAP medium (g.L^-1^)** | **HSM medium (g.L^-1^)** | **Johnson Medium (g.L^-1^)** | **Bristol Medium (g.L^-1^)** | ***Botryococcus* medium (g.L^-1^)** | ***Spirulina* medium (g.L^-1^)** | **M4N medium (g.L^-1^)** | **Modified Bold 3N medium (g.L^-1^)** | **Del Río Medium (g.L^-1^)** | **BG11-1 Medium (g.L^-1^)** | **Modified BG11 medium (g.L^-1^)** | Average Value (g/l) | **Average Value (mM)** |
| --- | --- | --- | --- | --- | --- | --- | --- | --- | --- | --- | --- | --- | --- |
| **NH_4_Cl** | 0.40 | 0.50 | - | - | - | - | - | - | - | - | - | 0.45 | **8.4** |
| **NaNO_3_** | - | - | - | 0.25 | 0.50 | 0.80 | - | 0.75 | - | 1.50 | 0.75 | 0.75 | **8.8** |
| **KH_2_PO_4_** | 0.056 | 0.323 | 0.035 | 0.03 | 0.028 | - | 1.25 | 0.325 | 0.236 | - | - | 0.285 | **2.1** |
| **CaCl_2_.2H_2_O** | 0.05 | 0.02 | - | 0.025 | 0.034 | 0.04 | - | 0.025 | 0.034 | 0.036 | 0.036 | 0.033 | **0.22** |
| **MgSO_4_·7H_2_O** | 0.1 | 0.02 | 0.25 | 0.03 | 0.06 | 0.05 | 0.004 | 0.075 | 0.04 | 0.04 | 0.075 | 0.078 | **0.316** |

**Additional Table 4: Individual Screen 2 media conditions.** Nutrient levels are coded as -1, 0 or 1, where 0 indicates the average value identified in Table 3, and -1 and +1 indicate the lower and higher concentrations tested for a given element (see materials and methods). CP indicates the centre point value (i.e. "0" settings for all ten nutrients varied in screen 2). 20 replicates of the centre point conditions were included in Screen 2 to provide a robust internal standard control. The centre point value corresponds to the basal nutrient concentration obtained from Screen 1).

**Notes on statistical variability**

Maximising the breadth of the multidimensional screening space is central to the screen design*.* For routine analysis it is therefore designed to operate using single trial wells as opposed to duplicates or triplicates, with the 20 CP controls providing a robust internal control for inherent variability and the estimation of error ranges. Statistical reproducibility however requires evaluation of operational errors both in terms of well-to-well variability (repeatability test) and run-to-run variability (reproducibility test).

The statistical evaluation of these errors was conducted using eight microalgae strains for *Screen 1* and three microalgae strains for *Screen 2.* Each species/trial combination was analysed in triplicate. Well-to-well repeatability was evaluated using the triplicate values of the highest growth rate values obtained for a given algae strain. Run-to-run reproducibility was evaluated using the highest growth rate values obtained for the triplicate TAP controls included in *Screen 1*.

The *Multivariate Item Analysis Cronbach’s Alpha* value was used to determine the degree of internal reliability and consistency of the triplicate data in these repeatability evaluations. The *Cronbach’s Alpha* value was calculated using *Additional Equation 1*

**Additional Equation 1:**


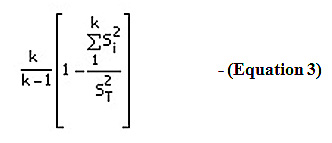


where S_i_^2^= growth rate variance of the i^th^ item, S_T_^2^= growth rate variance of the total scores, T = the total growth rate scores and k, the number of replicates or items in the analysis.

The *Omitted Items Cronbach’s Alpha* analysis is described in *Additional Equation 2*, and determines the level of correlation of the data between each of the three replicates by removing one of the replicate data points at a time. Replicate data with high correlativity will not show a significance difference in the *Omitted Items Cronbach’s Alpha* value.

**Additional Equation 2**


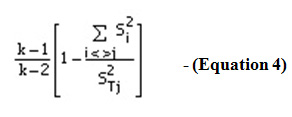


S_i_^2^= growth rate variance of the i^th^ item, S_T_^2^= growth rate variance of the total scores, S_Tj_^2^= growth rate variance of the total scores calculated after omitting the j^th^item, T = the total growth rate scores and k, the number of replicates or items in the analysis.

*Cronbach’s Alpha* values lie between 0 and 1 and the classification of the internal consistency of the data is as listed below:

> 0.9: Excellent internal consistency

0.8-0.9: Good internal consistency

0.7-0.8: Acceptable internal consistency

0.6-0.7: Questionable internal consistency

0.5-0.6: Poor internal consistency

< 0.5: Unacceptable internal consistency

The inter-replicate relationship trend and correlativity is graphically shown in the *Matrix Plots* in Additional Figure 5 (*Screen 1*) and Additional Figure 6 (*Screen 2*).

**Additional Figure 4** **- *Cronbach Alpha* analysis of Screen 1.2.** Each of the 8 strains were subjected to 60 Screen 1 nutrient conditions in triplicate (replicates = R1, R2 and R3). The individual highest growth rate in a specific cultivation media is represented by a red circle. The x- and y-axes display the highest growth rate, *µ* (h^-1^) of replicates 1, 2 and 3 and these are plotted in the following combinations: R1 vs. R2; R1 vs. R3; R2 vs. R3. In an ideal condition (i.e. all the replicates values are similar), the correlation line is a straight line with a slope value 1.0 (a perfect straight line). Data correlation line (blue line), showed positive correlation for all of the strains indicating high data consistency. Similar data trends between the replicates indicate excellent internal consistency (Cronbach alpha value >0.9).


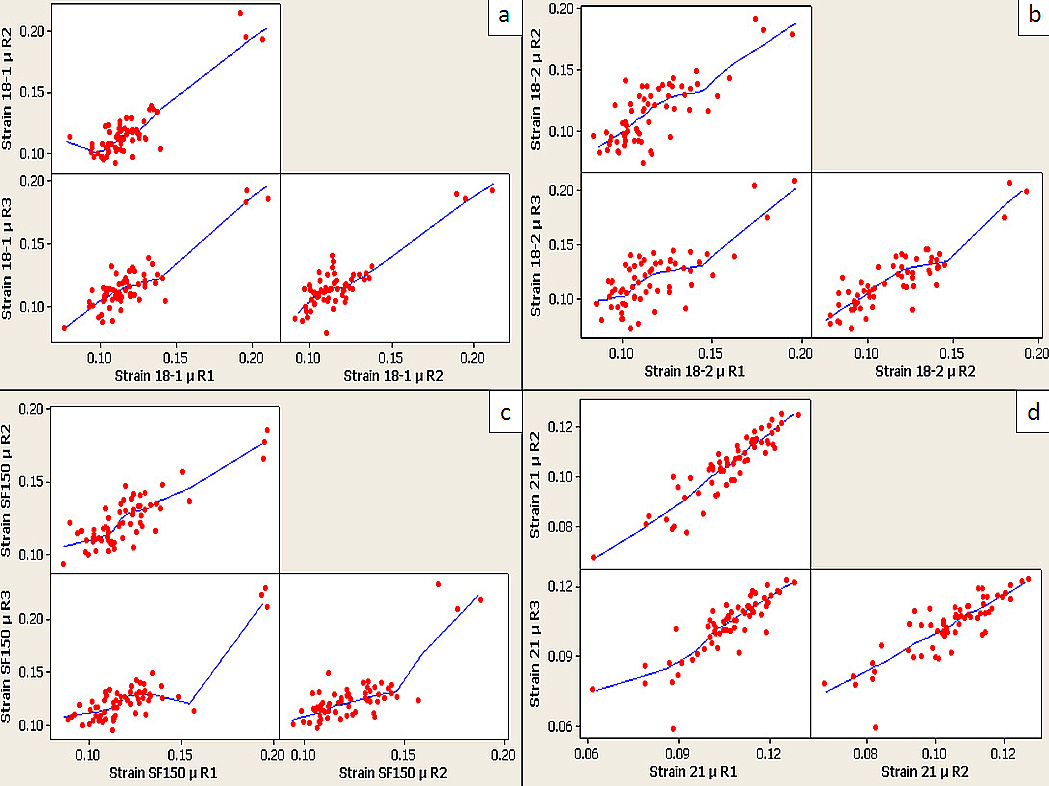

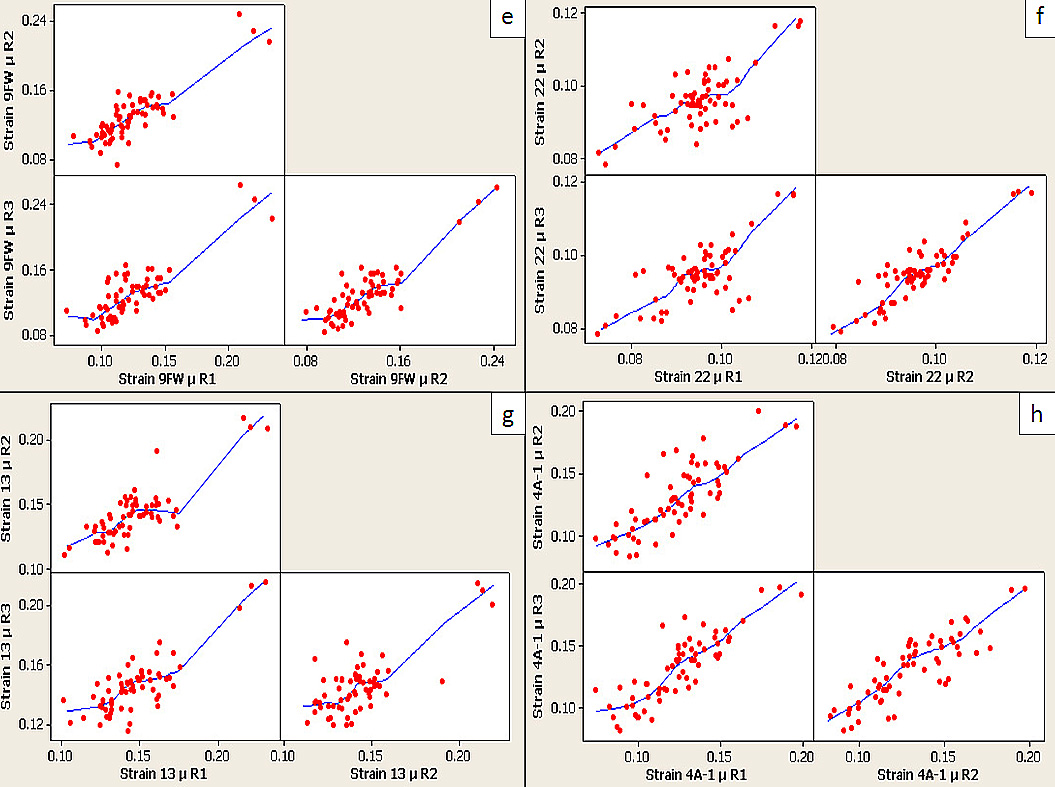


**Additional Figure 5** - **Cronbach analysis of Screen 2.** Each of the 3 strains were subjected to 180 Screen 2 nutrient conditions in triplicate (replicates = R1, R2 and R3). The individual highest growth rate in a specific cultivation media is represented by a red circle. The x- and y-axes display the highest growth rate, *µ* (h^-1^) of replicates 1, 2 and 3 and these are plotted in the following combinations: R1 vs. R2; R1 vs. R3; R2 vs. R3. Data correlation line (blue line), showed positive correlation for all of the strains indicating high data consistency. In an ideal condition (all the replicates values are similar), the correlation line is a straight line with a slope value 1.0 (a perfect straight line). Similar data trends between the replicates indicate excellent internal consistency (Cronbach alpha value >0.9).


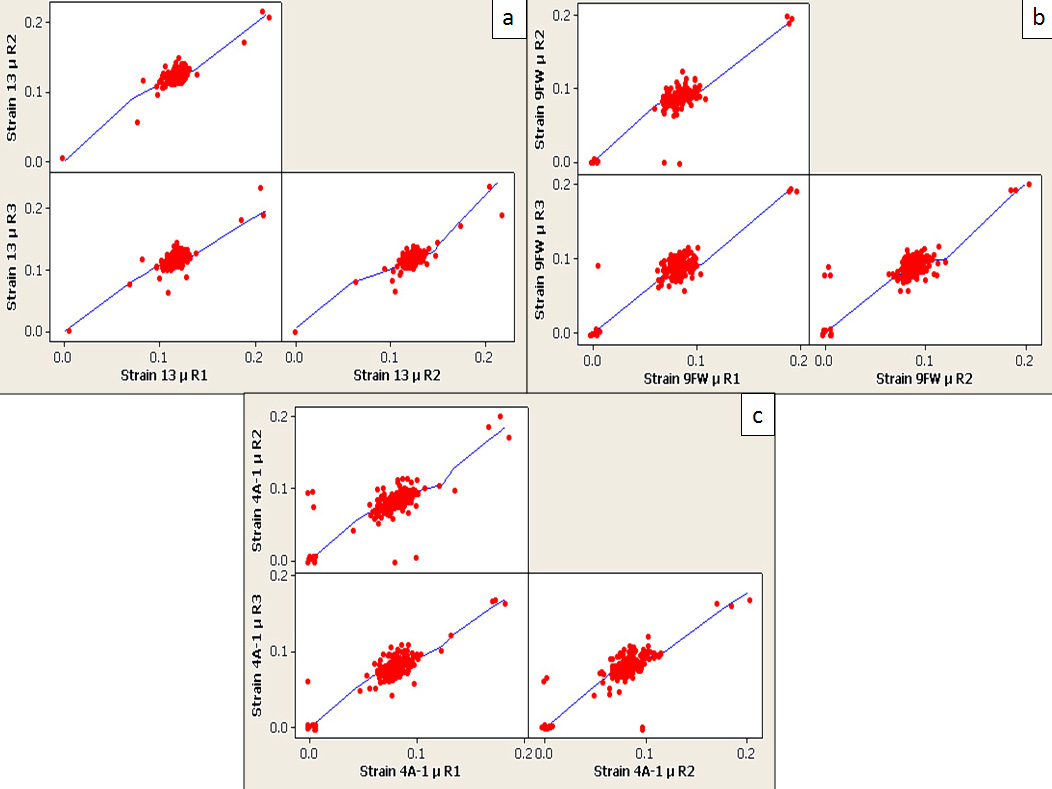


**Additional Figure 6:** **Analysis of the run-to-run reproducibility of the highest growth rate values obtained in screen 2.** The average highest growth rate for each of the microalgae strains in the TAP control medium is represented as a bar chart (data from 9 internal replicates; triplicates of 3 samples). Exp1 and Exp2 refer to two independent *Screen 1* trials. The average highest growth rate values obtained for each strain are shown above each corresponding bar chart. SD and Difference (%) denoted in the white boxes at the bottom of the bar charts represent the internal standard deviation and the inter-experiment percentage difference for each of the specific microalgae strains. The percentage differences ranged between 2.1 and 16.7%. Based on the average data of all of the strains, the inter-experiment percentage difference (reproducibility error was 8.1% ± 6.1 % (mean ± standard deviation).


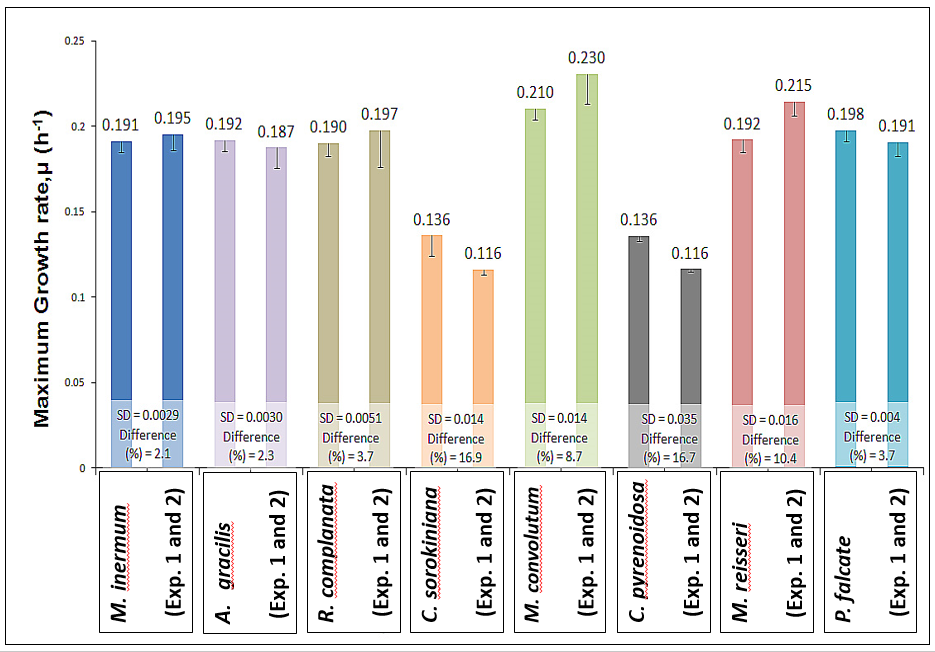


**References:**
